# Impact of Stepwise Balloon Angioplasty for De Novo Femoropopliteal Artery: A Retrospective Observational Study

**DOI:** 10.1002/hsr2.71366

**Published:** 2025-10-08

**Authors:** Takahiro Tokuda, Ryoji Koshida, Naoki Yoshioka, Takehiro Yamada, Akio Koyama, Kiyotaka Shimamura, Ryusuke Nishikawa, Yasuhiro Oba, Keisuke Hirano

**Affiliations:** ^1^ Department of Cardiology Nagoya Heart Center Nagoya Japan; ^2^ Department of Cardiology Hoshi General Hospital Koriyama Japan; ^3^ Department of Cardiology Ogaki Municipal Hospital Ogaki Japan; ^4^ Department of Cardiology Central Japan International Medical Center Minokamo Japan; ^5^ Department of Vascular Surgery Ichinomiya Municipal Hospital Ichinomiya Japan; ^6^ Department of Cardiology Shizuoka General Hospital Shizuoka Japan; ^7^ Department of Cardiovascular Medicine Kyoto University Graduate School of Medicine Kyoto Japan; ^8^ Department of Cardiology Toyohashi Heart Center Toyohashi Japan

**Keywords:** bail‐out stent, femoropopliteal artery, severe dissection, stepwise balloon angioplasty

## Abstract

**Background and Aims:**

Drug‐coated balloon use in the revascularisation of femoropopliteal (FP) lesions improves patency rates. However, conventional balloon angioplasty sometimes leads to severe artery dissection, which may be prevented using stepwise balloon angioplasty. This study aimed to examine the effects of stepwise balloon angioplasty, which is defined as a step‐up approach with a sequentially larger balloon size, on de novo FP artery disease.

**Methods:**

A retrospective analysis was performed using data collected from patients who underwent endovascular treatment for FP artery disease between August 2018 and December 2021 at eight institutions. During this period, 549 FP lesions were analyzed, and propensity score matching analysis was performed to compare stepwise and conventional balloon angioplasties. The prognostic value was assessed based on the angiographic dissection pattern, rate of bail‐out stenting, procedural complications, and rate of clinically driven target lesion revascularisation (CD‐TLR) within 1 year.

**Results:**

Propensity score matching was used to analyze 129 matched pairs of patients. The incidence of severe vessel dissection, defined as Type D or higher, and the rate of bail‐out stenting were significantly higher in conventional balloon angioplasty compared to stepwise balloon angioplasty (24.0% vs. 14.0%, *p* = 0.04; and 9.4% vs. 3.1%, *p* = 0.03, respectively). There were no significant differences between the two groups in procedural complications or the rate of CD‐TLR.

**Conclusion:**

Severe dissection and bail‐out stenting rates were lower after stepwise balloon angioplasty.

## Summary

- Drug‐coated balloon angioplasty improves femoropopliteal patency, but conventional single‐step dilation often causes severe dissections requiring bail‐out stenting.
- In this multicentre retrospective study, a stepwise balloon‐sizing strategy significantly reduced severe (type D or higher) dissections and the need for bail‐out stenting compared to conventional angioplasty.
- Incorporating stepwise angioplasty into routine femoropopliteal interventions may enhance procedural safety by lowering dissection and stent rates without increasing complications or CD‐TLR at 1 year.

## 1 Introduction

Drug‐coated balloons (DCBs) play a definitive role in the revascularisation of femoropopliteal (FP) disease. Previous studies have reported that using DCBs for the revascularisation of FP lesions leads to improved patency rates [1, 2, 3, 4]. However, predilation plays a crucial role in the application of DCB. Many studies have been published on the predilation of FP lesions. Tan et al. [5] reported that the use of long balloons for revascularisation in cases of chronic total occlusion (CTO) of FP lesions reduced the occurrence of severe dissection. Horie et al. [6] reported that prolonged balloon dilation is also effective in suppressing severe dissection. Furthermore, Sugihara et al. [7] reported that the superslow inflation method is a predictive factor for reducing severe dissection during balloon dilation. Despite the use of these methods, severe dissection after balloon angioplasty occurs occasionally.

To achieve adequate vascular expansion, appropriate predilation using an external elastic membrane (EEM)‐based balloon is necessary. However, conventional balloon angioplasty can cause severe dissection. To address this issue, we developed a stepwise balloon angioplasty technique that involves initially using a smaller balloon followed by gradual increases in the EEM‐based balloon size. To date, no reports have described the revascularisation of FP lesions using this stepwise approach. Therefore, we retrospectively examined the impact of stepwise balloon angioplasty as a predilation method for treating FP lesions across multiple centers.

## 2 Methods

### 2.1 Study Design and Patients

This was a multicenter retrospective cohort study that included patients undergoing endovascular treatment (EVT) for FP lesions across eight Japanese cardiovascular institutions.

The study was conducted at the following eight cardiovascular centers in Japan: Nagoya Heart Center (Nagoya), Hoshi General Hospital (Koriyama), Ogaki Municipal Hospital (Ogaki), Central Japan International Medical Center (Minokamo), Ichinomiya Municipal Hospital (Ichinomiya), Shizuoka General Hospital (Shizuoka), Kyoto University Hospital (Kyoto), and Toyohashi Heart Center (Toyohashi). A total of 549 consecutive patients (549 lesions) who underwent revascularisation with EVT for de novo FP lesions were included in this study. The inclusion and exclusion criteria was shown in Table 1.

**Table 1:** Inclusion and exclusion criteria.

Inclusion criteria
Age ≥ 18 years
Chronic, symptomatic lower limb ischemia (Rutherford 2–6)
Stenotic or occlusive lesion(s) located in the native femoropopliteal artery
Degree of stenosis ≥ 50% by visual angiographic assessment
Target vessel treated with drug‐coated balloon

Exclusion criteria
Target vessel with in‐stent restenosis
Cutting/scoring balloon use or atherectomy device use
Prior surgery or EVT of the femoropopliteal artery in the target limb to treat atherosclerotic disease
History of major amputation in the target limb
Life expectancy < 12 months because of other medical comorbid condition(s)
Asymptomatic patients
Pregnant and/or breast feeding
Current participation in another investigational drug or device clinical study that has not completed the primary endpoint at the time of enrollment
Presence of aneurysm in the target vessel
Acute ischemia and/or acute thrombosis of the femoropopliteal artery

Patients were divided into two groups based on the operator's discretion: a conventional balloon angioplasty group and a stepwise balloon angioplasty group. To account for baseline imbalances, we applied propensity score matching techniques with a 1:1 ratio to adjust for differences in baseline characteristics.

Demographic, angiographic, and procedural data were collected by independent researchers from each hospital and its database. Clinical follow‐up was scheduled at 1, 6, and 12 months, incorporating the ankle‐brachial index (ABI) measurement, duplex ultrasound, computed tomography (CT), or angiography, as available. Information was extracted from institutional databases and patient records, and when necessary, supplemented through direct communication with patients or their healthcare providers. The study protocol was approved by the Ethics Committees or Review Boards of the eight centers and conducted in accordance with the tenets of the Declaration of Helsinki. Due to the retrospective nature of the study, the requirement for written informed consent from patients was waived.

### 2.2 Intervention

Depending on the complexity of the lesion, an ipsilateral antegrade or contralateral crossover approach was selected at the operator's discretion. For the ipsilateral antegrade approach, a 6Fr sheath, 6Fr Destination (Terumo, Tokyo, Japan), or 6Fr Parent Sheath (Medikit, Tokyo, Japan) was introduced through the ipsilateral common femoral artery. For the contralateral crossover approach, a 6Fr Destination, 6Fr Parent Sheath, or 6Fr Sheathless PV (Asahi Intec, Aichi, Japan) was inserted through the contralateral femoral artery. After sheath insertion, unfractionated heparin was administered to achieve an activated clotting time greater than 250 s. A 0.014‐/0.018‐/0.035‐inch guidewire was used to cross the FP lesions, depending on lesion characteristics and operator preference. If the target lesion was a CTO, the procedure was performed by advancing the guidewire through the intraplaque route as far as possible under guidance from angiography, intravascular ultrasound, or duplex ultrasound.

After the guidewire crossed the lesion, intravascular ultrasound was performed to assess the size of the EEM, and a balloon was selected accordingly. The balloon was then inflated at nominal pressure for 2–3 min. In the stepwise balloon angioplasty group, a balloon 1–2 mm smaller than the EEM was initially selected and inflated for 1 min. Subsequently, inflation was performed for 2–3 min using a balloon the same size as the EEM. If the EEM was not an integer, for example, if the EEM was 5.5 mm or smaller, a 5‐mm balloon was used in both groups. If the EEM was 5.6 mm or larger, a 6‐mm balloon was used. After balloon dilation, the operator had discretion to use a DCB or deploy a stent as the final device. Antiplatelet therapy with aspirin (100 mg daily), clopidogrel (75 mg daily), or cilostazol (100 mg twice daily) was initiated at least 1 week before EVT and continued for at least 1 month for bare‐metal stents or 3 months for drug‐eluting stents. At least one antiplatelet drug was administered to all patients after EVT.

### 2.3 Endpoints

The primary endpoints were defined as final dissection grade and bail‐out stent rate.

Secondary endpoints included post‐procedural ABI, procedural complications, primary patency of the FP artery at 1 year, and clinically driven target lesion revascularization (CD‐TLR) at 1 year.

### 2.4 Definitions

Procedure success was defined as residual stenosis of < 30% without a suboptimal result. Dissection severity was graded according to the National Heart, Lung, and Blood Institute classification system [8]. Final dissection was evaluated before using the final devices for FP lesions. Chronic limb‐threatening ischemia includes a broader and more heterogeneous group of patients with varying degrees of ischemia, which may delay wound healing and increase the risk [9]. Severe calcification was classified as peripheral artery calcification scoring system Grade 3 or higher [10]. The procedural complications included death, pseudoaneurysm, hematoma/hemorrhage at the puncture site, distal embolization, wire perforation, residual guidewire, vascular rupture, arteriovenous fistula, and transfusion. Poor run‐off is defined as a condition in which zero or only one below‐the‐knee artery remains patent. Primary patency was defined as the absence of restenosis of the treated vessel or revascularisation that remained patent. CD‐TLR was defined as repeated EVT at the initial target lesion if > 50% stenosis was found at follow‐up. Restenosis was determined if follow‐up imaging or physiological testing revealed > 50% luminal narrowing (by angiography, duplex ultrasound, or CT) or if the duplex‐derived peak systolic velocity ratio exceeded 2.4. A drop in resting ABI ≥ 0.20 was also considered indicative of restenosis [11].

### 2.5 Statistical Analysis

The data gathered in this study were analyzed using statistical methods previously published in guidelines and clinical research [12, 13].

To mitigate confounding, we generated propensity scores through logistic regression models that incorporated all clinical and lesion‐related covariates. All baseline characteristics (age, sex, body mass index, hypertension, diabetes mellitus, dyslipidemia, chronic kidney disease, hemodialysis, coronary artery disease, chronic obstructive pulmonary disease, cerebrovascular disease, atrial fibrillation, current smoking, cilostazol use, ambulatory status, reference diameter, lesion length, CTO, severe classification, run‐off, pre‐ABI, balloon reference, balloon length, and inflation time) were included as covariates. Matching was performed with a caliper width of 0.20 on the logit of the propensity score [14]. Data are presented as numbers and percentages, mean ± standard deviation, or median (interquartile range). Categorical variables were compared between groups using the *χ* ^2^ test or Fisher's exact test, as appropriate. Continuous variables were compared using the *t*‐test or the Mann–Whitney *U*‐test.

Primary patency and CD‐TLR of the FP artery were compared using the Kaplan–Meier method, followed by a log‐rank test after propensity score matching.

Two‐sided statistical significance was set at a *p*‐value of <0.05. All statistical analyses were performed using JMP software (version 14.0.2; SAS Institute Inc., Cary, NC, USA).

## 3 Results

**Figure 1:**
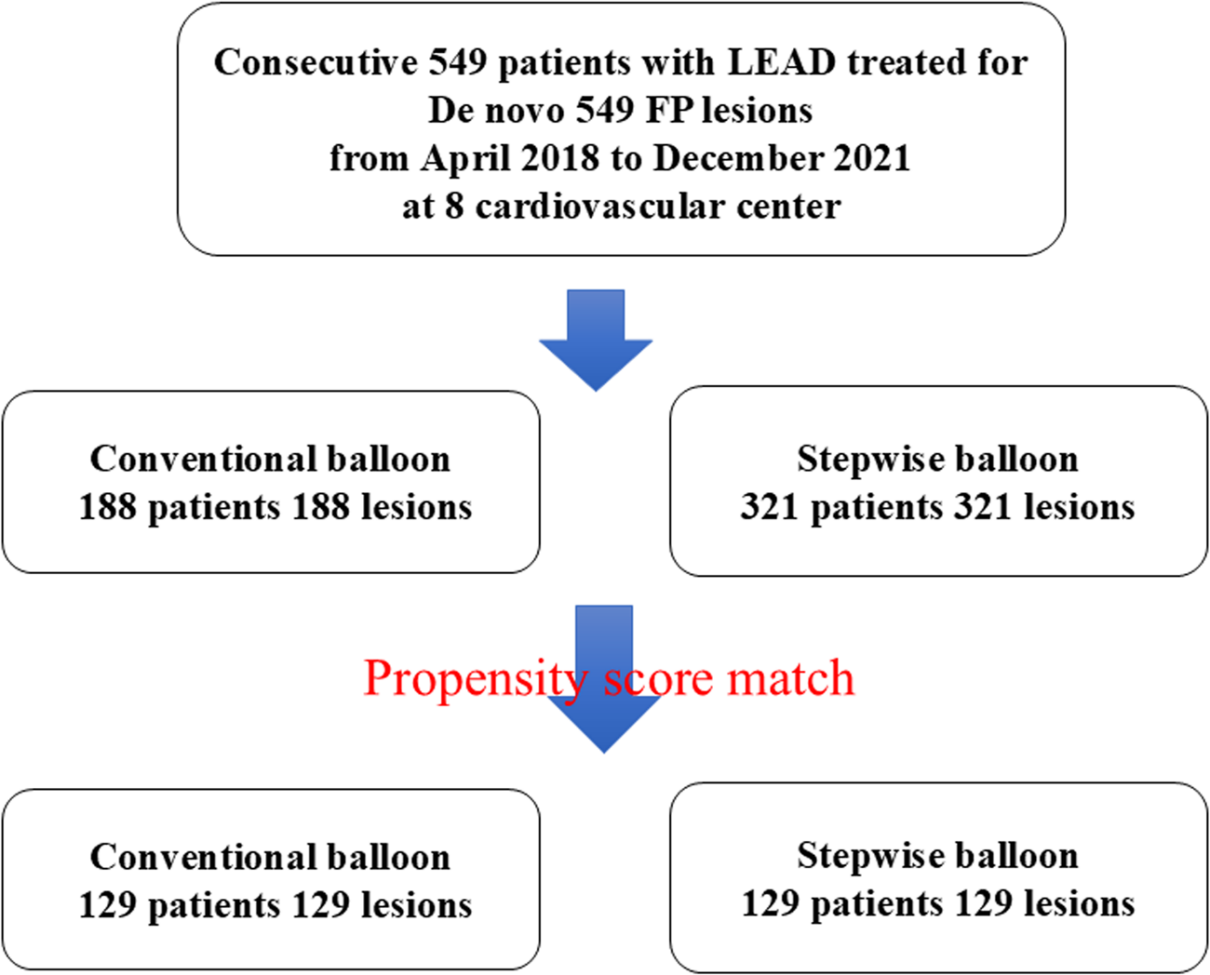
Patient flowchart. FP, femoropopliteal; LEAD, lower‐extremity artery disease.

The patient inclusion flowchart is shown in Figure 1. Of the 549 patients included in the analysis, 188 underwent conventional balloon angioplasty and 321 underwent stepwise balloon angioplasty. After propensity score matching, 129 pairs of patients were included in the analysis. The baseline patient and lesion characteristics are summarized in Tables 2 and 3, respectively. Before matching, several significant differences were observed in patient and lesion characteristics. A lower rate of patients with dyslipidemia (44.2% vs. 58.0%; *p* = 0.003), a higher rate of patients with coronary artery disease (60.5% vs. 51.9%; *p* = 0.04), higher cilostazol usage (24.3% vs. 14.9%; *p* < 0.001), and a lower ABI (0.57 ± 0.20 vs. 0.61 ± 0.19; *p* = 0.02) were observed in the stepwise balloon angioplasty group than in the conventional balloon angioplasty group. Higher prevalence of CTO (33.3% vs. 20.2%; *p* < 0.001), longer lesion length (137.9 ± 89.5 mm vs. 102.8 ± 87.3 mm; *p* < 0.001), and a higher rate of severely calcified lesions (22.4% vs. 14.4%; *p* = 0.02) were also more characteristic of the stepwise balloon angioplasty group than the conventional balloon angioplasty group (Tables 2 and 3). In the matched populations, no significant differences were observed in baseline patient, lesion, or procedural characteristics between the two groups.

**Table 2:** Patient characteristics.

	**Before matching**				**After matching**		
	**Conventional group**	**Stepwise group**	**SMD**	***p* value**	**Conventional group**	**Stepwise group**	**SMD**
No	188	321			129	129	
Age (years)	74.5 ± 9.9	74.5 ± 9.5	0.5	0.96	74.8 ± 10.5	74.3 ± 9.8	4.9
Male, *n* (%)	123 (65.4)	206 (64.2)	2.5	0.78	78 (60.5)	82 (63.6)	6.4
Body mass index (kg/m^2^)	22.6 ± 3.3	22.1 ± 3.5	14.1	0.09	22.3 ± 3.4	21.9 ± 3.6	11.4
Hypertension, *n* (%)	156 (83.0)	264 (82.2)	16.3	0.83	114 (88.4)	106 (82.2)	16.3
Diabetes mellitus, *n* (%)	117 (62.2)	200 (62.3)	0.2	0.99	86 (66.7)	83 (64.3)	5.0
Dyslipidemia, *n* (%)	109 (58.0)	142 (44.2)	31.6	0.003	73 (56.6)	66 (51.2)	10.8
Hemodialysis, *n* (%)	71 (37.8)	141 (43.9)	12.2	0.17	52 (40.3)	60 (46.5)	12.5
Chronic kidney disease, *n* (%)	95 (50.5)	183 (57.0)	13.0	0.16	78 (60.5)	79 (61.2)	1.4
Coronary artery disease, *n* (%)	126 (51.9)	185 (60.5)	17.3	0.04	72 (55.8)	69 (53.5)	4.6
Cerebral vascular disease, *n* (%)	28 (14.9)	51 (15.9)	4.3	0.75	22 (17.1)	20 (15.5)	4.3
Current smoking, *n* (%)	42 (22.3)	78 (24.3)	4.7	0.61	26 (20.2)	29 (22.5)	5.6
Cilostazol use, *n* (%)	28 (14.9)	87 (27.1)	30.0	< 0.001	28 (21.7)	26 (20.2)	3.7
Chronic limb threatening ischemia, *n* (%)	40 (21.3)	62 (19.3)	5.0	0.59	27 (20.9)	26 (20.2)	1.7
Ambulatory, *n* (%)	160 (85.1)	285 (88.8)	11.0	0.23	109 (84.5)	108 (83.7)	2.2
Pre procedural ABI	0.61 ± 0.19	0.57 ± 0.20	20.5	0.02	0.61 ± 0.2	0.61 ± 0.8	5.4

Abbreviations: ABI, ankle brachial index; SMD, standardized mean difference.

**Table 3:** Lesion and procedural characteristics.

	Before matching				After matching		
	Conventional group	Stepwise group	SMD	***p* value**	Conventional group	Stepwise group	SMD
No	188	321			129	129	
Chronic total occlusion, *n* (%)	38 (20.2)	107 (33.3)	29.6	< 0.001	35 (27.1)	32 (24.8)	5.2
Reference diameter (mm)	5.2 ± 0.8	5.2 ± 0.8	1.3	0.84	5.3 ± 0.8	5.2 ± 0.8	1.1
Lesion length (mm)	102.8 ± 87.3	137.9 ± 89.5	54.0	< 0.001	118.6 ± 95.6	126.1 ± 91.5	5.7
Poor run off, *n* (%)	89 (47.3)	151 (47.0)	0.6	0.98	62 (48.1)	65 (50.4)	4.6
Severe calcification, *n* (%)	27 (14.4)	72 (22.4)	19.9	0.02	27 (20.9)	31 (24.0)	7.2
Pre‐balloon reference (mm)	4.9 ± 0.8	4.7 ± 1.0	22.1	0.07	4.8 ± 0.9	4.6 ± 0.8	2.5
Pre‐balloon length (mm)	92.6 ± 63.1	133.1 ± 78.5	57.6	< 0.001	113.6 ± 75.0	110.8 ± 67.3	3.9
Inflation time (sec)	139.6 ± 65.0	218.1 ± 89.7	84.0	< 0.001	162.8 ± 72.1	165.0 ± 80.0	2.9

Abbreviation: SMD, standardized mean difference.

The key adjusted baseline characteristics were as follows: average age, 74 years; male sex, 60.5%–63.6%; diabetes, 64.3%–66.7%; hemodialysis, 40.3%–46.5%; CTO, 24.8%–27.1%; and lesion length, 119–126 mm (Tables 2 and 3).

The rate of final dissection Grade D or higher and the bail‐out stent incidence were significantly lower in the stepwise balloon angioplasty group than in the conventional balloon angioplasty group, whereas the post‐procedural ABI and procedural complications were not significantly different between the two groups (Table 4).

**Table 4:** Intervention results.

	Conventional group	Stepwise group	***p* value**
	129	129	
Final dissection ≧ D, *n* (%)	31 (24.0)	18 (14.0)	0.04
Post‐procedural ABI	0.85 ± 0.17	0.84 ± 0.17	0.73
Bail out stent rate, *n* (%)	12 (9.4)	4 (3.1)	0.03
Procedural complications, *n* (%)	3 (2.3)	5 (3.9)	0.47

Abbreviation: ABI, ankle brachial index.

**Figure 2:**
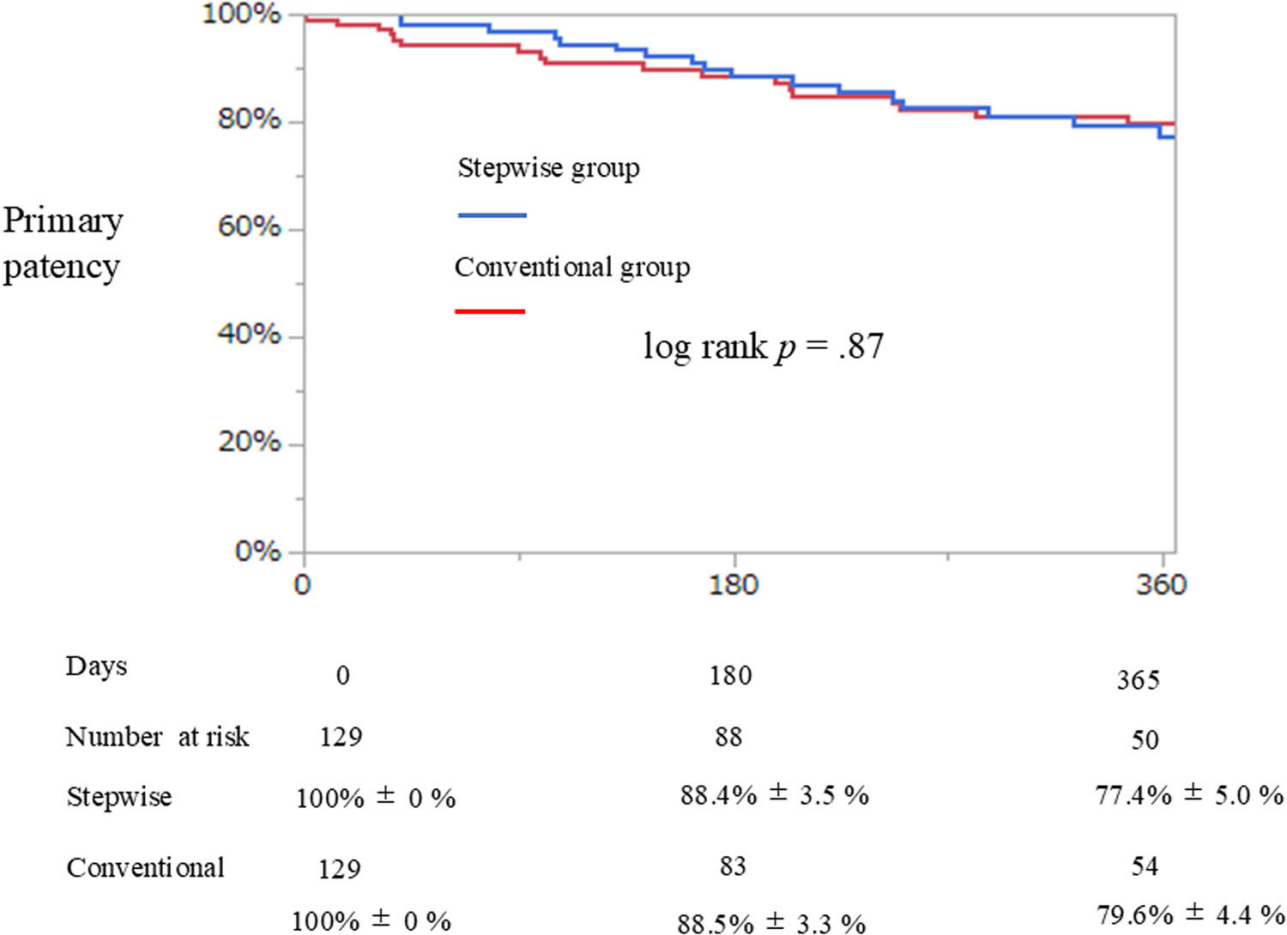
Kaplan‐Meier curve of primary patency.

**Figure 3:**
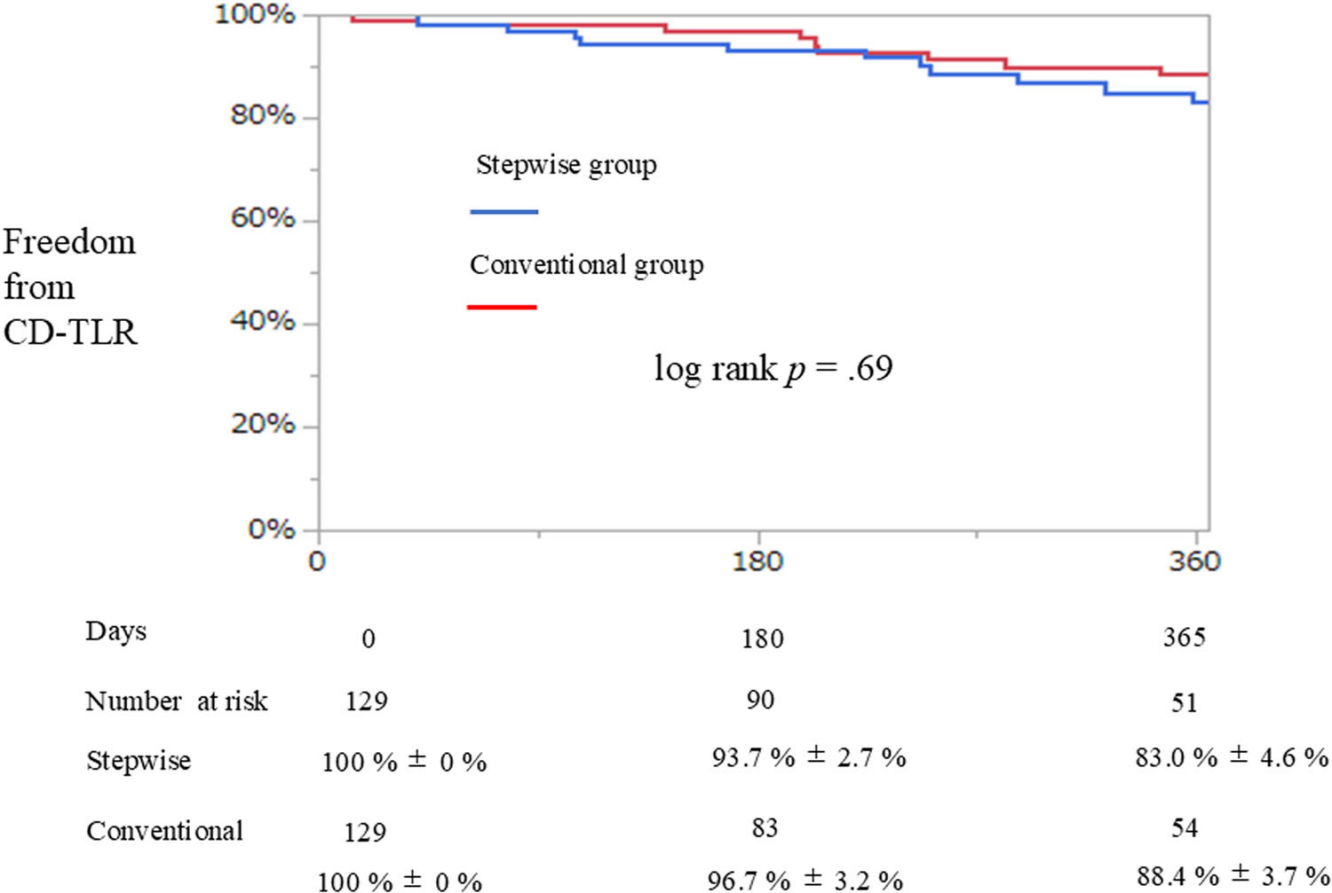
Kaplan‐Meier curve of freedom from CD‐TLR. CD‐TLR, clinically driven target lesion revascularization.

Loss of primary patency was observed in 74 patients (28.7%) over a median follow‐up period of 361 days (IQR: 91–731 days). Of these, 53 patients (20.5%) underwent EVT during the follow‐up. In addition, no significant differences were observed in the primary patency of the FP artery or CD‐TLR between the two groups (Figures 2 and 3).

## 4 Discussion

In this study, we elucidated two key findings regarding the use of stepwise balloon angioplasty for treating FP lesions. First, the rate of severe vascular dissection after lesion dilation was lower in the stepwise balloon angioplasty group. Second, this method reduced the frequency of bail‐out stenting. DCB has emerged as the first‐line treatment for FP lesions in contemporary clinical practice. It is well established that maximizing the effectiveness of DCB requires achieving adequate vascular dilation while minimizing vascular dissection. According to a Japanese expert consensus document, selecting an appropriately sized balloon based on the EEM diameter is crucial for sufficient dilation and effective vessel expansion [15]. With the appropriate balloon size, inflation pressure, and inflation time, optimal vessel preparation could be achieved if residual stenosis or severe dissection does not occur [16]. However, severe dissection sometimes occurs after dilation of FP lesions with conventional balloon angioplasty, and it has been reported that the patency rate decreases with the onset of type D or higher vascular dissections, which means less dissection and larger dilation are mandatory for better patency [17, 18, 19]. Techniques the operators can control in cases of CTO include ensuring that the guidewire passes as centrally as possible and selecting a long balloon that fully covers the lesion [5, 20, 21]. The occurrence of severe dissection is considered to be influenced by the guidewire passage route and plaque morphology. According to single‐center reports, achieving central wiring can help suppress the incidence of severe dissection; however, the relationship between plaque morphology and severe dissection has not been demonstrated [20, 22]. Furthermore, specific plaque morphological characteristics, such as specific calcification grade, fibrous cap thickness, and lipid core size, may alter vessel wall stress distribution during balloon inflation and thus contribute to dissection formation. However, the mechanistic link between plaque morphology and severe dissection has not been fully elucidated and warrants further investigation. Balloons sized to the EEM can exert pressure on vulnerable plaque areas, potentially causing severe vascular dissection. To mitigate this risk, a stepwise approach was devised. This method involves initially using a smaller‐diameter balloon to uniformly reshape the plaque, allowing sufficient dilation when subsequently employing an EEM‐sized balloon. This technique achieves adequate expansion while minimizing the risk of severe dissection. Using the stepwise approach may help prevent severe dissection and bail‐out stenting. However, no significant differences were observed in primary patency or CD‐TLR, which was attributed to the use of the same final balloon size in both groups. Additionally, other factors, including prior EVT history, lesion distribution and morphology, and the type of DCB, also influence primary patency and CD‐TLR [23]. Given the higher rate of bail‐out stent implantation in the conventional balloon angioplasty group despite severe angiographic dissections occurring in 24.0% versus 14.0% of cases (*p* = 0.04) and stents deployed in only 9.4% versus 3.1% of cases (*p* = 0.03), leaving 14.6% of high‐grade dissections untreated, it is possible that the actual rates of primary patency and CD‐TLR in this group may be somewhat lower than observed because untreated dissections may undergo vessel recoil or adverse remodeling.

In this study, scoring balloons, cutting balloons, and atherectomy devices were excluded to focus on the impact of stepwise balloon angioplasty. The study had a retrospective design and utilized propensity score matching to balance patient demographics and lesion characteristics. Using this method, we effectively validated our hypotheses. Specifically, we demonstrated that stepwise balloon angioplasty significantly reduces severe dissection and the need for bail‐out stenting. We believe that this stepwise approach may enhance the potential use of DCB as the final device for treating FP lesions. To rigorously validate our findings, future multicenter prospective randomized trials are necessary.

### 4.1 Study Limitations

Several limitations should be acknowledged. The matched sample size was modest, and the retrospective design without independent core‐lab adjudication may have introduced bias; therefore, the dissection grade could not be measured objectively. Third, we did not analyze the impact of differences between semi‐compliant and non‐compliant balloons. Fourth, mortality was not included as a parameter in the present study; therefore, we were unable to assess its potential impact on primary patency or CD‐TLR. Fifth, the stepwise balloon angioplasty protocol requires the use of an additional balloon per lesion, which may increase procedural supply costs. Although this technique significantly reduced bail‐out stent implantation rates, we did not collect unit‐cost data for balloons, stents, or associated procedural time. Consequently, a formal cost‐effectiveness analysis, incorporating the price differential between an additional balloon, as well as procedure duration and resource use, was not performed in this study. Future prospective studies should include detailed economic evaluations to determine the net budgetary impact of the stepwise strategy. Finally, all participants were Japanese, which could limit extrapolation to populations with different clinical or anatomical characteristics. Further prospective studies with larger sample sizes are warranted to confirm our results in other populations.

## 5 Conclusion

Stepwise balloon angioplasty prevented severe vessel dissection and bail‐out stenting for the dilation of FP lesions.

## Author Contributions

**Takahiro Tokuda:** conceptualization, data curation, investigation, methodology, project administration, writing – original draft, writing – review and editing. **Ryoji Koshida:** conceptualization, data curation, investigation. **Naoki Yoshioka:** data curation, writing – review and editing. **Takehiro Yamada:** writing – review and editing, data curation. **Akio Koyama:** writing – review and editing. **Kiyotaka Shimamura:** writing – review and editing. **Ryusuke Nishikawa:** writing – review and editing. **Yasuhiro Oba:** writing – review and editing, data curation, supervision, investigation. **Keisuke Hirano:** data curation, writing – review and editing.

## Conflicts of Interest

The authors declare no conflicts of interest.

## Transparency Statement

The lead author Takahiro Tokuda affirms that this manuscript is an honest, accurate, and transparent account of the study being reported; that no important aspects of the study have been omitted; and that any discrepancies from the study as planned (and, if relevant, registered) have been explained.

## Data Availability

The data that support the findings of this study are available on request from the corresponding author. The data are not publicly available due to privacy or ethical restrictions.
